# Factors associated with medication self-management performance in the “Ability to Self-administer Medication in Non-demented In-hospital Patients” (ABLYMED) study

**DOI:** 10.1186/s12877-026-07077-7

**Published:** 2026-02-02

**Authors:** Anneke Luegering, Robert Langner, Stefan Wilm, Thorsten R. Doeppner, Dirk M. Hermann, Helmut Frohnhofen, Carla Stenmanns, Janine Gronewold

**Affiliations:** 1 Hospital Pharmacy, University Hospital Düsseldorf, Düsseldorf, Germany; 2 Institute of Systems Neuroscience, University Hospital Düsseldorf, Düsseldorf, Germany; 3 Institute of Neuroscience and Medicine (INM-7: Brain and Behaviour), Research Centre Jülich, Jülich, Germany; 4 Institute of General Practice, Centre for Health and Society (chs), University Hospital Düsseldorf, Düsseldorf, Germany; 5 Department of Neurology, University Hospital Giessen, Giessen, Germany; 6 Research Institute for Health Sciences and Technologies (SABITA), Istanbul Medipol University, Istanbul, Türkiye; 7 Department of Anatomy and Cell Biology, Medical University of Varna, Varna, Bulgaria; 8 Department of Neurology, University of Göttingen, Göttingen, Germany; 9 Department of Neurology and Center for Translational Neuro- and Behavioral Sciences (C-TNBS) University Hospital Essen, University Duisburg-Essen, Essen, Germany; 10 Department of Orthopedics and Trauma Surgery, Heinrich-Heine-University, Düsseldorf, Germany; 11 Faculty of Health, University Witten Herdecke, Witten, Germany

**Keywords:** Medication self-management, Older adults, Cognitive impairment, Manual dexterity

## Abstract

**Background:**

Older people often suffer from medication management problems due to multimorbidity, polypharmacy and medication complexity. Because of frequent discrepancies between self-reported and actual abilities to self-administer prescribed medications, these problems often go unnoticed. To secure adequate medication management for effective pharmacotherapy, it is important to understand which factors possibly influence medication self-management performance. As part of a large-scale study on the “Ability to Self-administer Medication in Non-demented In-hospital Patients” (ABLYMED), we addressed this question by assessing medication- and patient-related factors with a possible influence on medication self-management in 100 patients ≥ 70 years of age regularly taking ≥ 5 different medicines autonomously.

**Methods:**

Medication management performance was assessed in five different placebo dosage forms via standardized video-based evaluation expressed in an overall performance rating score. Data from 57 patients (median age 78, Q1; Q3 = 73;82 years) could be used for subsequent analyses. To analyze which factors are associated with performance in medication management, we calculated correlations of different medication- (e.g. complexity, adequacy in older age) and patient-related factors (e.g. adherence, cognition, motor abilities, burden of disease, age, sex) with self-administration performance and included all factors with significant correlations in a stepwise multivariable linear regression model.

**Results:**

We observed significant correlations between the video-based measure of performance in medication management and the following patient-related factors: cognition, manual dexterity, functional state, activities of daily living and age. When performing multivariable linear regression stepwise selection, age and ZVT-G (test method for the construct cognition, especially information processing speed) remained in the model.

**Conclusions:**

Our research suggests that cognitive and motor impairments in old age have a negative impact on medication management. Higher age and lower information processing speed are associated with poorer ability to self-administer medication. Further investigations will address the consequences of these findings for patient trainings, medication prescription procedures and careful control for problems in medication management and external help.

**Clinical trial registration:**

DRKS00025788, date of registration: 07/09/2021.

## Background

Older people and multimorbid patients frequently obtain pharmacotherapies [1]. Due to the multimorbidity of older patients, many different medicines are often required to treat patients according to guidelines for specific diseases [2, 3]. Patients aged 80 years and older have an average of three diagnoses [4], and the common diseases of old age, such as arterial hypertension, diabetes and heart failure, usually require a combination of medicines. Therefore, polypharmacy is common in this patient group [5, 6], which implies the daily use of five or more medicines [5, 7]. The number of medicines a patient needs to administer is one factor that determines the complexity of a medication regimen. This is because each additional medicine often brings its own dosage and administration instructions, which can make the regimen more difficult to manage. Other factors include the number of different dosage forms and times of administration [8]. These complexity factors increase the level of difficulty in medication management, with special impact on older people.

Due to the often complex nature of medication regimens in combination with the functional and cognitive decline associated with ageing, the daily medication self-management is challenging for older patients [2, 6]. The ageing process is associated with cognitive and motor impairments [2]. With regard to cognitive function, studies have demonstrated a decline in working memory and episodic memory in ageing individuals [2]. Functional impairments can encompass a range of symptoms, such as difficulty swallowing, impaired vision, weakness in hand strength, and loss of fine motor skills [9, 10]. As a result, older people are at a higher risk for medication-related problems and non-adherence. However, medication management problems are often not addressed or not recognized in routine care, especially in patients living independently [11, 12]. Therefore, efforts should be made to identify patients with medication management problems, because medication-related problems may lead to patient harm and increasing healthcare costs [13].

Previous research has attempted to assess medication self-management skills in order to identify patients at risk of medication management problems, developing tools to detect cognitive and physical barriers to successful medication management [14, 15]. Medication management skills have been assessed in a variety of ways. These studies utilised the concept of adherence as a metric to ascertain the construct validity of medication management performance [14]. For example, the Medication Management Instrument for Deficiencies in the Elderly (MedMaIDE) [16], which was identified in a scoping review of Badawoud et al. as the instrument that most closely matched the proposed characteristics of an ideal instrument for assessing medication self-management skills, assesses three domains by questioning and observation: knowledge of medication, ability to take medication, and knowledge about procurement [14, 16]. In our ABLYMED study, we assessed medication management performance via video-based evaluation of patients’ medication management skills in addition to the MedMaIDE [17]. When medication management problems are identified, they can first be addressed by individual patient trainings to improve medication management or by modification of the prescribed medication regimens to reduce complexity or replace modes of administrations which pose a challenge for the patient. In a systematic review Jin et al. described, that e.g. reviewing and optimizing medication schedules, using social support and better integration of medication management tasks in the daily routines were key elements to improve medication self-management capacity. In most studies from this review, medication self-management capacity was assessed via interview [18]. To secure adequate medication management, which is a prerequisite for effective pharmacotherapy, it is important to understand which factors influence medication self-management performance. If modifiable, these factors can be additionally addressed in patient trainings or in medication prescription. If not modifiable, patients exhibiting these characteristics can be carefully controlled for problems in medication management and offered external help like outpatient nursing services. Consequently, we evaluated patient- and medication-related factors for their association with observed medication management performance. Therefore, with our research question, we would like to address a gap in literature. To the best of our knowledge the extant literature has not yet addressed factors that possibly influence medication self-management performance based on an objective measurement as employed in the ABLYMED study.

## Methods

### Sample

The cross-sectional single-center observational ABLYMED study included 100 non-demented patients from the Department of Orthopedics and Trauma Surgery and the Department of Vascular and Endovascular Surgery of the University Hospital Duesseldorf, aged ≥ 70 years who regularly took ≥ 5 different medicines autonomously [19]. Patients were continuously recruited from September 2021 to April 2022. Patients were enrolled in the study provided they met the predefined inclusion and exclusion criteria and gave written informed consent. Inclusion criteria were age 70 years and above and regularly taking five or more different medicines autonomously. Exclusion criteria were dementia diagnosis (ICD-10-code F00-F03), legal care, insufficient ability to self-administer medication at home, insufficient ability to communicate, poor vision, agraphia, alexia, instable clinical condition, permanently bedridden and palliative condition. Incomplete data collection did not lead to exclusion. Data collection consisted of demographic factors, video-based evaluation of medication self-management performance and an assessment of potential factors that influence medication self-management performance.

Of the 100 patients enrolled in the ABLYMED study, 67 agreed to be videotaped and 57 patients completed performance in all dosage forms. Therefore, data from 57 patients could be used for subsequent analyses. The most common reason for missing data on medication management performance was patients not agreeing to the video-recordings, in some cases recording had to be discontinued because a disturbance-free environment could not be ensured. The study was reviewed and approved by the local Ethics Committee at the Medical Faculty of the Heinrich Heine University Düsseldorf (reference number 2021–1435). Prior to participation in the study, written informed consent was obtained from all patients.

### Study protocol

#### Video-based evaluation of medication self-management performance

Participants had to demonstrate their medication handling skills via standardized self-administration tasks involving five different placebo dosage forms while being filmed. The assessment focused on the fundamental handling procedures of each dosage form, as the primary aim was to systematically evaluate patients’ basic practical skills before considering more complex medication management tasks. The video recordings were then analyzed and evaluated by two independent raters to quantify self-administration performance [17, 19, 20].

Patients were instructed to perform standardized self-administration tasks involving five different placebo dosage forms (tablets: tablet removal from a blister pack and a tablet tube, cutting a tablet and filling a pill organizer, eye-drops: opening an one-dose ophtiole dispenser, oral drops: opening a child-resistant dropper bottle and aiming ten drops on a teaspoon, pens: removal of tow caps, dialing 12 units and injection into a ball and patches: unpacking, peeling of the protective liner and applying onto the skin) independently of the prescribed medication. The patients’ hands were videorecorded during the self-administration tasks. The video recordings, five per patient (one for each dosage form), were made by AL (pharmacist) and independently evaluated by the two raters JG (psychologist and epidemiologist) and TD (neurologist). The evaluation of the video recordings was based on a standardized assessment form, rating rules and training developed by experts in geriatrics. The raters evaluated each step of medication administration for each dosage form on a 5-point Likert-type rating scale (5 = not possible, i.e., practical assistance needed or interruption; 4 = severe difficulties, i.e., execution hardly possible or success of therapy at risk; 3 = moderate difficulties, i.e., execution significantly slowed down; 2 = mild difficulties, i.e., execution slightly slowed down; 1 = no difficulties, i.e., correct and fluid execution). In addition, in some cases the assessment form allowed a choice between correct and incorrect (1 = correct and 2 = incorrect) (see Table 1). The scores of each step of medication administration were summed separately for each dosage form (tablets: range 4–17, eye-drops: range 1–5, oral drops: range 3–12, pen: range 4–20 and patch: range 3–15) and across all dosage forms (total sum score considering performance across all dosage forms: range 15–69) [17, 20].

**Table 1:** Assessment form for the video ratings of medication self-management performance [17]

**Tablets**		
White tablet removal from the blister pack	no difficulties	1
	mild difficulties	2
	moderate difficulties	3
	severe difficulties	4
	not possible	5
Blue tablet removal from the tablet tube	no difficulties	1
	mild difficulties	2
	moderate difficulties	3
	severe difficulties	4
	not possible	5
Cutting the blue tablet	no difficulties	1
	mild difficulties	2
	moderate difficulties	3
	severe difficulties	4
	not possible	5
Correctly filling the pill organizer	Correct	1
	Incorrect	2
Sum score		
Eye-drops		
Open the one-dose ophtiole dispenser	no difficulties	1
	mild difficulties	2
	moderate difficulties	3
	severe difficulties	4
	not possible	5
Sum score		
Oral drops		
Open the child-resistant dropper bottle	no difficulties	1
	mild difficulties	2
	moderate difficulties	3
	severe difficulties	4
	not possible	5
Targeting at the teaspoon	no difficulties	1
	mild difficulties	2
	moderate difficulties	3
	severe difficulties	4
	not possible	5
Correct number of drops (n=10) on the teaspoon	Correct	1
	Incorrect	2
Sum score		
Pen		
Remove the transparent cap of the pen	no difficulties	1
	mild difficulties	2
	moderate difficulties	3
	severe difficulties	4
	not possible	5
Remove the green cap of the needle	no difficulties	1
	mild difficulties	2
	moderate difficulties	3
	severe difficulties	4
	not possible	5
Dialing in the right dose (12 units)	no difficulties	1
	mild difficulties	2
	moderate difficulties	3
	severe difficulties	4
	not possible	5
Injection into a ball	no difficulties	1
	mild difficulties	2
	moderate difficulties	3
	severe difficulties	4
	not possible	5
Sum score		
Patch		
Unpack the patch	no difficulties	1
	mild difficulties	2
	moderate difficulties	3
	severe difficulties	4
	not possible	5
Peeling off the protective liner	no difficulties	1
	mild difficulties	2
	moderate difficulties	3
	severe difficulties	4
	not possible	5
Apply it onto the skin	no difficulties	1
	mild difficulties	2
	moderate difficulties	3
	severe difficulties	4
	not possible	5
Sum score		
Total sum score		

### Assessment of potential factors of influence on medication self-management performance

Medication- and patient-related factors with a possible influence on medication self-management skills were identified based on previous expert interviews and literature reviews. Tables 2 and 3 show these factors together with information on how they were measured and which parameters were evaluated. For further information, see our study protocol [19].

**Table 2:** Medication-related factors and their assessment

Factor/Construct	Test method	Parameter
number of different medicines regularly taken assessed via interview and medication schedule	Number of medicines	Number
number of medicines classified as D (should be avoided in older people; omit first and reviewed/find alternatives) in the FORTA list	Number of medicines FORTA (Fit for The Aged) D [4]	Number
anticholinergic burden	ACB-Score (anticholinergic burden score) for German prescribers [21]	anticholinergic burden score
complexity of the medication regimen, defined by number of different dosage forms, dosage frequency, as well as specific directions concerning the administration	MRCI-D (Medication Regimen Complexity Index) German version [8]	Index

**Table 3:** Patient-related factors and their assessment

Factor/Construct	Test method	Parameter
indirect adherence	MARS-D (Medication Adherence Report Scale) German version [22]	total score
manual dexterity and cognitive capacity	Timed Test of Money Counting [23]	time (seconds)
higher-level cognitive function	Clock-drawing test [24]	score
temporal orientation and short-term memory	Six-item screener [25]	score
information processing speed	ZVT-G (Trail Making Test for older subjects) [26]	time (seconds)
temporal orientation	Time estimation Task one: estimation of one minute Task two: estimation of length of hospitalization in days	task one: magnitude of deviation (seconds) task two: ‘correct’ or ‘incorrect’
visual-spatial skills	Drawing interlocking pentagons [27]	‘correct’ or ‘incorrect’
manual dexterity and complex visual-motor coordination	Peg-Board time right hand, Peg-Board drops right hand, Peg-Board time left hand, Peg-Board drops left hand Lafayette Instrument^®^, Model 32,025, https://lafayetteevaluation.com/products/grooved-pegbo ard	time (seconds), number of drops
functional state, total muscle mass	Pinch strength right, Pinch strength left, Grip strength right, Grip strength left	strength (kilogram)
skeletal muscle mass	Circumferences of the mid-upper arm, Circumferences of the leg calf, Triceps skinfold thickness [28]	circumference/thickness (centimeters)
more complex activities of daily living	IADL (instrumental activities of daily living) [29]	score
basic activities of daily living	Katz-Index [30]	score
frailty	CFS (Clinical Frailty Scale) [31]	score
nutritional status	MNA-SF (Mini Nutritional Assessment - Short Form) [32]	score
sarcopenia	SARC-F (Strength, Assistance with walking, Rise from a chair, Climb stairs and Falls) [33]	score
tendency to fall	History of falls	yes or no
burden of disease	Charlson Comorbidity Index^a^ [34]	score
medication management skills	MedMaIDE^b^ [16]	score
chronological age	Age	years
biological sex (self-reported)	Sex	male or female
education	ISCED 2011 (International Standard Classification of Education 2011) [35]	level

^a^ The calculation is based on 19 underlying diseases, each of which is assigned a different weighting. The CCI also includes points for the age of the patients, which are deliberately omitted here, because age is assessed alone

^b^ The Medication Management Instrument for Deficiencies in the Elderly: In contrast to the original work, we did not use a total deficiency score, where higher values indicating superior deficiency. Instead, a point-based system was used, with one point allocated for each fulfilled item. The points system ranges from 0 to 13, with higher values indicating less deficiency

### Statistical analysis

To analyze which factors possibly influence medication self-management performance, we calculated correlations of different medication- and patient-related factors with the total sum rating score representing medication management performance across all dosage forms. Subsequently, all factors with significant correlations (*p* < 0.05) were included in a stepwise multivariable linear regression model plus the factors age and sex. The correlation between the continuous total sum score describing the ability to self-administer medication across all dosage forms and nominal patient- or medication-related factors was analyzed by means of Eta-coefficient (η). The correlation between the continuous total sum score and continuous and ordinal patient- or medication-related factors was analyzed by means of Spearman´s Rho correlation coefficient. The multivariable linear regression stepwise selection method was conducted with the following criteria: probability of F-value for enter ≤ 0.05, probability of F-value for remove ≥ 0.10. The statistical analyses were conducted using SPSS 28 for Windows (IBM Corporation, Armonk, NY, USA). All statistical tests were two-tailed, and p-values below 0.05 were considered statistically significant. Since the present study constitutes an exploratory investigation, the significance level was not adjusted for the number of tests.

## Results

The median age of this analysis sample was 78 years (Q1;Q3 = 73;82 years), and 49% were female. The median number of different medicines regularly taken was 9 medicines (Q1;Q3 = 7;12), the median Charlson Comorbidity Index was 1,0 (Q1;Q3 = 1,0;2,8). The median adherence score MARS-D was 25 (Q1;Q3 = 23;25), the median IADL score was 7,5 (Q1;Q3 = 5,0;8,0) and the median Six-item screener score of our patients was 5,5 (Q1;Q3 = 4,0;6,0). The median total sum score representing medication management performance across all dosage forms of this analysis sample was 23,0 (Q1;Q3 = 20,3;28,5).

Table 4 shows the correlations between medication self-management performance across all dosage forms and the medication-related factors for number of medicines, for number of medicines FORTA D, for ACB-Score and for MRCI-D. For all medication-related factors, there were no significant correlations.

**Table 4:** Correlations between total sum score and medication-related factors

Type of factor	Factor	Correlation coefficient	*p*-value
Medication-related	Number of medicines	−0.01	0.921
	Number of medicines FORTA D	−0.05	0.733
	ACB-Score	0.13	0.340
	MRCI-D	−0.03	0.840

Data are shown as Spearman´s Rho coefficient and p-value, significant correlations are highlighted in bold

Table 5 shows the correlations between total sum score and patient-related factors. The correlating factors relate to cognition, manual dexterity and complex visual-motor coordination, functional state, geriatric assessment parameter, medication management skills, and biological age. Significant correlations were found, especially in the domain of cognition (Timed Test of Money Counting: Spearmans’s Rho = 0.45; Clock-drawing test: 0.36; ZVT-G: 0.38) and for age (Spearman’s Rho = 0.33). There were no significant correlations with the patient-related factors adherence, skeletal muscle mass, burden of disease, sex and education.

**Table 5:** Correlations between the total sum score and patient-related factors

Type of factor	Factor	Correlation coefficient	*p*-value
Patient-related (adherence)	MARS-D	0.05	0.726
Patient-related (cognition)	**Timed Test of Money Counting**	**0.45**	**0.001**
	**Clock-drawing test**	**0.36**	**0.009**
	Six-item screener	−0.10	0.455
	**ZVT-G**	**0.38**	**0.004**
	**Time estimation**		
	**Task one**	**0.31**	**0.020**
	Task two	0.12*	0.378
	Drawing interlocking pentagons	0,07*	0.607
Patient-related (manual dexterity and complex visual-motor coordination)	Peg-Board time right	0.16	0.275
	**Peg-Board drops right**	**−0.29**	**0.043**
	Peg-Board time left	0.25	0.078
	Peg-Board drops left	0.02	0.880
Patient-related (functional state)	**Pinch strength right**	**−0.30**	**0.027**
	**Pinch strength left**	**−0.34**	**0.011**
	Grip strength right	−0.25	0.066
	**Grip strength left**	**−0.27**	**0.042**
Patient-related (skeletal muscle mass)	Circumferences of the mid-upper arm	−0.11	0.430
	Circumferences of the leg calf	−0.16	0.262
	Triceps skinfold thickness	0.08	0.635
Patient-related (geriatric assessment)	**IADL**	**−0.30**	**0.026**
	Katz-Index	−0.27	0.050
	Frailty CFS	0.19	0.180
	MNA-SF	0.00	0.990
	SARC-F	0.18	0.210
	history of falls	0.03*	0.835
Patient-related (burden of disease)	Charlson Comorbidity Index	−0.07	0.592
Patient-related (medication management skills)	**MedMaIDE**	**−0.33**	**0.012**
Patient-related (patient characteristics)	**Age**	**0.33**	**0.013**
	Sex	0.20*	0.129
	Education	−0.03	0.830

Data are shown as Spearman´s Rho or Eta-coefficient (*) and p-value, significant correlations are highlighted in bold

Performing stepwise linear regression with all significant variables from Tables 4 and 5 plus sex, the factors age (b = 0.25, *p* = 0.032) and processing speed using ZVT-G (b = 0.07, *p* = 0.016) remained in the model to predict patients’ ability to self-administer medication in all dosage forms (see Table 6). In a sensitivity analysis not including sex, results remained exactly the same.

**Table 6:** Results of stepwise multivariable linear regression for the criterion variable total sum score with all significant correlations from Tables 4 and Table 5 plus sex

	Regression coefficient b (non-standardized)	*p*-value
Constant	0.91	0.916
Processing Speed/ZVT-G (sec.)	0.07	0.016
Age (years)	0.25	0.032

## Discussion

Analyzing the association of a variety of patient-related and medication-related factors with medication management performance measured via an objective video-based performance measure by two raters in older multimorbid patients reporting autonomous medication management, we found that older age and lower information processing speed, as measured by the ZVT-G, was associated with the ability to self-administer medication.

In a narrative review conducted by the HIOPP-6 research group aiming at the identification of factors increasing complexity of medication treatment and nonadherence, which in turn can lead to worse medication self-management performance, six superordinate categories were identified, i.e., dosage forms, product characteristics, dosage schemes, additional instructions, patient characteristics and process characteristics [36]. Patient characteristics with impact on the adherence were age, not being partnered or married or not having support in medication handling, female sex, level of education, poor numeracy, low health literacy, cognitive and physical limitations. For the patient characteristic age, the existing literature demonstrates that a younger age (younger than 65 years) as well as a higher age (older than 84 years) are associated with nonadherence [36–39]. A study by Curtin et al. revealed that compared to older dialysis patients (68 patients > 65 years), younger dialysis patients (67 patients ≤ 65 years) exhibited a significantly higher incidence of noncompliance, tracked by a medication event monitoring system, with their antihypertensive medications (47% vs. 42% of noncompliance) and their phosphate binders (80% vs. 65% of noncompliance) [37]. Similar to our results showing that higher age analyzed as a continuous variable was associated with lower ability to self-administer medication, Chapman et al. observed that higher age was associated with nonadherence (defined by prescriptions sufficiently covering < 80% of days) to antihypertensive and lipid-lowering therapy in an older Medicare-eligible population ≥ 65 years. Especially age ≥ 85 years was associated with nonadherence compared with age 65–74 years in this retrospective cohort study, even though this age group only represented < 4% of the total cohort [38]. For the patient characteristic cognition, Valdeoriola et al. reported that in a cohort of 418 patients with Parkinson´s disease, cognitive impairment was associated with lower level of adherence (2.1 times higher likelihood to take treatment incorrectly). Adherence was measured by Morisky-Green test, the measurement of cognitive impairment was not further described [40]. In a sample more similar to our study cohort and using also comprehensive cognitive testing, Insel et al. observed that in 95 community-dwelling older adults 63–93 years who self-administered their medication, only a composite score of executive function and working memory predicted medication adherence (examined over 8 weeks for one prescribed medicine by use of an electronic medication monitoring cap) in multivariable regression, while age was no significant predictor [41]. In our study, we also used different tests to assess cognition. Several significant correlations were identified between medication management performance and the cognitive tests including the Timed Test of Money Counting, Clock-drawing test and ZVT-G, while in the multivariable model ZVT-G measuring information processing speed remained significantly related to medication management performance. A study by Anderson et al. showed a significant correlation between test performance in the Trail Making Test Part B and 30-day readmission in hospital in older patients with independent medication management, with the risk being particularly elevated in patients who were taking a minimum of seven medications [42].

Turner et al. assessed associations between adherence (measured by comparing the amount of medication used with the amount of medication prescribed) and cognitive impairment, physical function, and depression assessed in a geriatric in-home assessment in 100 community-dwelling adults ≥ 65 years of age who were - in contrast to our study - identified with self-neglect via Adult Protective Services. The lowest adherence level was significantly associated with lower physical function levels assessed by a Physical Performance Test which covers motor function of the upper and lower extremities. The low adherence group (< 29%) had the lowest Physical Performance Test scores (mean ± SD = 12.6 ± 6.3), followed by the high adherence group (> 86%, 15.6 ± 5.1) and the moderate adherence group (29–86%, 16.4 ± 4.1). In contrast to our study, cognition (assessed by Mini-Mental State Examination (MMSE)) and basic and instrumental activities of daily living were not significantly associated with adherence [43]. Regarding physical function, we mainly found correlations between medication management performance and grip and pinch strength. In the discussion of the literature above, adherence was the main measure of medication management in most studies, whereas in our study medication management performance was measured. The employment of a direct medication management performance measure is a distinctive feature of our study. Frequently, adherence is utilized as a metric to ascertain the construct validity of medication management performance [14]. Adherence describes the alignment between patient´s behavior and the agreed recommendations from the prescriber. It also encompasses factors such as procuring medication, understanding the benefits and risks of medication, and managing medication. However, medication management capacity depends on functional limitations and is not knowingly influenced by the patient [44]. In comparison our method of video-based observation of medication handling for placebo dosage forms reflects the capacity for specific medication management performance without including other factors that are encompassed in adherence. An established instrument assessing medication management performance is MedMaIDE. It covers the three domains knowledge of medications, how to take medications, and procurement. In comparison to our method of video-based observation of medication handling for placebo dosage forms, the instrument is based on questioning and observing patients about their own medication. Therefore, the correct application is not checked for all dosage forms. However, our method does not cover the domains knowledge and procurement, which are included in the MedMaIDE score. In 50 community-living adults aged ≥ 65 years taking at least one prescribed medication autonomously [16], medication adherence was assessed by MedMaIDE and via 30-day pill-count to show concurrent validity. Further, cognitive function was assessed via MMSE and functional status via ADLs and IADLs to evaluate divergent validity of MedMaIDE. The correlation for the association between the total MedMaIDE deficiency score and pill count compliance was − 0.52, implying a modest concurrent validity. Divergent validity was shown by lower correlations between the MedMaIDE total deficiency score and both the ADL (*r* = 0.13) and MMSE scores (*r*=−0.44). The modest negative correlation between MedMaIDE and MMSE, similar to our study results, shows that lower cognitive function is associated with worse medication management. However, in contrast to our study, this study did not use comprehensive cognitive testing but applied the MMSE, which represents a cognitive screening tool. We did not find significant correlations between adherence and medication management skills, although the methods of measurement were different (interview versus pill count, interview is more susceptible to bias by patients not willing to report low adherence). The MedMaIDE sample is comparable to the ABLYMED sample concerning age, number of medicines and independent medication management. However, the studies differ in terms of the constructs measured. In the ABLYMED study, adherence of the patients was assessed using the MARS D, while cognitive function was evaluated comprehensive cognitive testing. The MedMaIDE utilised pill count to determine adherence and MMSE to assess cognition [16]. Of note, the Wakuya project discovered an association between medication management reported by family members of non-demented older adults and medication-related semantic memory, but not with general cognitive and executive functions. Consequently, the assessment of the semantic memory would have been interesting; however, this approach was not pursued in the ABLYMED study [45].

The identification of factors which potentially influence medication self-management performance has important clinical implications. While age is a non-modifiable risk factor, age-related decline in information-processing speed might be mitigated by psychological training within certain limits [46, 47]. Further, patients identified as vulnerable for low medication self-management abilities because of high age and/or low information processing speed, can be regularly monitored regarding their ability to self-administer medication. Their medication regimens should be kept as simple as possible, and in case problems in medication self-administration are identified, individualized trainings can be provided on how to take medication correctly. If problems cannot be solved by these measures, external help can be provided, for example via nursing service.

The HIOPP-6 research group, which identified factors associated with medication complexity based on the idea that higher complexity can lead to worse medication management performance as described above [36], later developed key questions to identify problems in medication management based on their review, patient opinions and expert opinions [48]. These key questions were additionally validated in a small sample of patients in a pharmacy by practical medication management tasks with placebo medication similar to our study. Contrary to our study, only complex dosage forms were chosen in this pilot phase. Based on the key questions, optimization measures were proposed and evaluated in a group of 126 patients from general practitioners [49]. According to the evaluation from the patient perspective, the tailored optimization measures based on the integration of the key questions were rated more beneficial to reduce or mitigate complexity of medication treatment than non-tailored measures not based on the key questions or routine care. Unfortunately, medication management performance was not assessed after optimization.

In the ABLYMED study, we compared patients’ objective video-based medication management performance with their self-reported performance in an interview. It turned out, that the interview alone did not reveal the objective medication management skills. There were patients overestimating as well as patients underestimating their performance [50]. It is therefore questionable whether the key questions are sufficient to identify patients at risk for medication management problems, even though they were much more detailed than the questions in the ABLYMED study [50–52]. In a next phase of the ABLYMED project, we thus plan to combine the video-based performance measure of medication management, questions regarding problems with medication management, comprehensive geriatric assessment, key questions from the HIOPP-6 project and different optimization techniques and patient trainings to further evaluate clinical relevance of assessing and improving medication management in older people.

Despite the clinical impact of our current findings to the field, we must also acknowledge limitations. We only included hospitalized patients in the study, which limits generalizability to other populations. The setting could have a negative effect on medication management performance. A comparison with patients in general practice is useful to quantify the influence of the hospital setting. Such a comparison is in progress. We only analyzed a rather small sample of 57 patients (*n* = 100 patients included in the ABLYMED study, *n* = 67 performed video-recorded medication management and *n* = 57 completed all dosage forms). Even though the present study constitutes an exploratory investigation, the small sample size did not allow for thorough control of type 1 error, possibly leading to false positive results. Studies with larger sample sizes are needed to confirm our observation and guide clinical decision making. We did not consider the fact that some patients had more practice with the dosage forms included in their own medication. However, in the ABLYMED sample all patients regularly used tablets, but only 24% used eye drops, 22% used pens, 7% used oral drops and 2% used patches. Finally, we must note that patients with dementia diagnosis were excluded using ICD-10 codes F00-F03 in previous medical reports. Unfortunately, we could not take other diagnostic criteria into account and were not able to perform more comprehensive neuropsychological testing in the study context. Thus, it cannot be ruled out that patients with mild dementia, who had not received a dementia diagnosis so far, were included in our cohort.

Further, the distinction between medication- and patient-related factors remains open to discussion. Since we did not manipulate these factors systematically, a causal interpretation of the relationship between the factors and medication management performance might not be possible. However, even if these associations are not causal, they are important for the identification of patients at risk for problems in medication management.

## Conclusion

Our study investigated factors associated with medication self-management performance based on our video-based performance measure of the self-administration of placebo medication in different dosage forms in older independently living people with polypharmacy. Our results suggest that older age and slower information processing speed, as measured by the ZVT-G, which is short and easy to administer in clinical routine, are associated with poorer ability to self-manage medication. Further research will highlight the implications of these findings for geriatric assessment and medication prescribing.

## Data Availability

The datasets used and/or analysed during the current study are available from the corresponding author on reasonable request.
